# Design of a Human Rhinovirus-14 3C Protease-Inducible Caspase-3

**DOI:** 10.3390/molecules24101945

**Published:** 2019-05-21

**Authors:** Hanna J. Wagner, Wilfried Weber

**Affiliations:** 1 Faculty of Biology, University of Freiburg, Schänzlestraße 18, 79104 Freiburg, Germany; 2 Signalling Research Centres BIOSS and CIBSS, University of Freiburg, Schänzlestraße 18, 79104 Freiburg, Germany

**Keywords:** caspase, cleavage, enzyme, mutation, protease, protein engineering, synthetic biology

## Abstract

The engineering of enzymes for the purpose of controlling their activity represents a valuable approach to address challenges in both fundamental and applied research. Here, we describe and compare different design strategies for the generation of a human rhinovirus-14 (HRV14) 3C protease-inducible caspase-3 (CASP3). We exemplify the application potential of the resulting protease by controlling the activity of a synthetic enzyme cascade, which represents an important motif for the design of artificial signal transduction networks. In addition, we use our engineered CASP3 to characterize the effect of aspartate mutations on enzymatic activity. Besides the identification of mutations that render the enzyme inactive, we find the CASP3-D192E mutant (aspartate-to-glutamate exchange at position 192) to be inaccessible for 3C protease-mediated cleavage. This indicates a structural change of CASP3 that goes beyond a slight misalignment of the catalytic triad. This study could inspire the design of additional engineered proteases that could be used to unravel fundamental research questions or to expand the collection of biological parts for the design of synthetic signaling pathways.

## 1. Introduction

Proteases are important tools for biological research and applications, such as proteomics, structural biology, or the biotechnological manufacturing of proteins. For example, the modification of proteins by means of solubility tags or affinity tags is commonly used to ensure proper expression yields and to facilitate protein purification. Since these additional protein domains or peptide tags might interfere with downstream processes, protease-based protocols have been established for their efficient removal [1]. Importantly, some endoproteases show stringent specificity for their target sequence. Among them, the tobacco etch virus protease (TEV) or the human rhinovirus serotype 14 (HRV14) 3C protease are well-characterized and usually used for tag- and protein-domain removal [1,2,3].

In recent years, the usage of proteases has been extended to new fields of research. Several site-specific proteases have been engineered to function as signal receptors and transducers in synthetic biological approaches [4,5,6,7]. Examples include the chemically induced reconstitution of split proteases [4,8], the activation of autoinhibited proteases [5], the optogenetic control of protease conformation [9,10,11], or the control of post-translational protein degradation and gene transcription [6,12]. Whereas the engineering of split or autoinhibited proteases requires careful evaluation of structural properties and often library-based selection and optimization, naturally occurring zymogens offer a more straightforward way towards custom-designed, inducible proteases. Inactive precursors adopt their active form by, for instance, the removal of inhibitory peptide units or cleavage events that induce conformational rearrangements [13,14]. As a consequence, the location or time of enzymatic action can be precisely controlled. This principle plays a crucial role in various physiological processes such as digestion, blood coagulation, complement activation, or programmed cell death. In the latter case, the activation of caspase (cysteinyl aspartic protease) cascades is key to controlling and inducing apoptosis and inflammatory events [15]. The dimerization of initiator caspases enables their autoproteolytic activation and the subsequent cleavage and activation of executioner caspases, which mediate programmed cell death through the cleavage of hundreds of cellular substrates. Dysregulation of caspases, and thus cell death, is associated with severe human diseases such as cancer, autoimmune diseases, and neurodegeneration [16]. Hence, the development of approaches to precisely control the activation of caspases represents a promising approach to study the physiological and pathological molecular processes involved in cell death and inflammation [8,9]. Moreover, inducible caspases offer novel therapeutic approaches to, for example, eliminate virus-infected cells [17].

Here, we describe strategies for the generation of human rhinovirus-14 (HRV14) 3C protease-inducible caspase-3 (CASP3) variants. The 3C protease shows high sequence specificity for the octapeptide sequence LEVLFQGP (hereafter referred to as 3C protease cleavage site, 3CS), making it an ideal candidate for precisely cleaving and activating engineered, 3CS-containing CASP3 variants. We exploit the domain structure of the proenzyme (pro-CASP3), which comprises a pro-domain and large (p17) and small (p12) subunits. Cleavage of pro-CASP3 by initiator caspases leads to the formation of the p17 (17 kDa) and p12 (12 kDa) fragments that assemble in a heterotetrameric conformation with two active sites at opposite ends of the enzyme [18]. Notably, pro-CASP3 is capable of autoactivation. However, under normal cellular conditions, this is strictly inhibited by a “safety catch” tripeptide within the p12 domain of pro-CASP3 [19]. By contrast, pro-CASP3 autoproteolytically adopts its active form when overexpressed or produced in bacterial cells [20]. Inspired by previous studies to control the activation of CASP3 [8,17,20], we eliminated the endogenous cleavage site for activation and tested different strategies for introducing a 3C protease recognition sequence. The CASP3/3C protease duo offers the potential to be used in combination with other proteases such as orthogonal potyvirus proteases. We therefore applied the resulting 3C protease-inducible CASP3 for the design of a short proteolytic cascade. Moreover, we studied effects of CASP3 mutations on cleavage efficiency and catalytic activity.

## 2. Results

**Figure 1:**
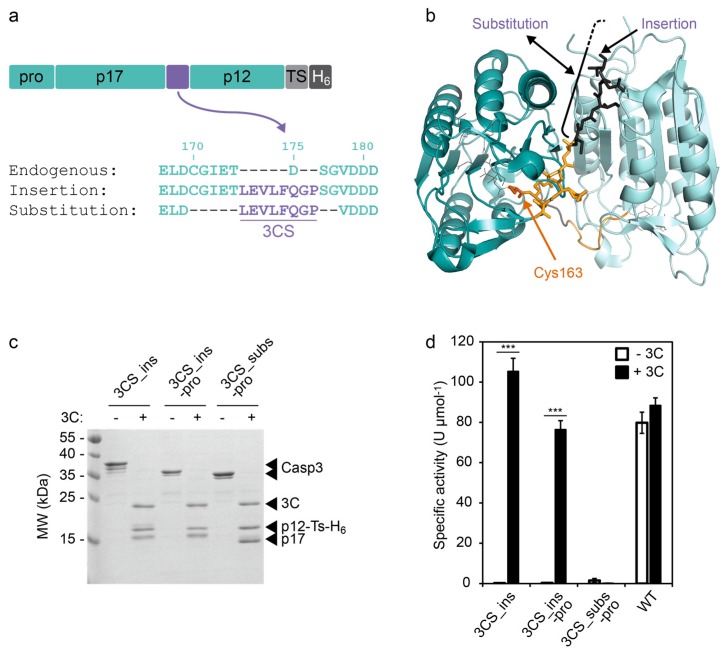
Design and testing of a 3C protease-inducible CASP3. (**a**) Incorporation of a 3C protease cleavage site (3CS) between the large (p17) and small (p12) subunits of CASP3. 3CS was inserted at position 175 (termed “insertion”), thereby deleting the endogenous aspartate for autoprocessing. Alternatively, residues 170–177 were substituted with 3CS (termed “substitution”). A hexa-histidine tag (H_6_), which can optionally be removed via a TEV protease cleavage site (TS), was fused to the C-terminus of the caspases. (**b**) Structure of mature CASP3 (PDB ID: 3EDQ [22]). The two p12/p17 heterodimers are colored in dark and light cyan, respectively. The site of 3CS insertion is indicated by a black arrow. The substituted amino acids 170–174 are colored in black (residues 175–177 are not included in the structure). The yellow and black colored residues constitute loop L2, which contains the catalytic cysteine-163 (marked in orange). The figure was generated using PyMOL [23]. (**c**) Cleavage of CASP3 constructs by 3C protease. The 0.2 mg/mL CASP3 constructs 3CS_ins (5.3 µM), 3CS_ins-pro (5.7 µM), and 3CS_subs-pro (5.8 µM) were incubated in the absence (−) or presence (+, 2.4 µM) of 3C protease (3C) for 4 h at RT and analyzed using SDS-PAGE. (**d**) Activity of CASP3 constructs. The indicated constructs were incubated in the absence (−3C) or presence (+3C, 2.4 µM) of the 3C protease in the same way as described in (c) (the WT concentration was 5.4 µM). The specific activity of CASP3 in 1:100-diluted samples was determined by a colorimetric assay using a synthetic DEVD-*p*NA substrate. Mean values of three replicates +/− s.e.m. are shown. Statistics: unpaired t-test; *** *p* < 0.005. Construct abbreviations: 3CS_ins, CASP3 with inserted 3CS; 3CS_ins-pro, 3CS_ins with deleted prodomain; 3CS_subs-pro, CASP3 with substituted cleavage site and deleted prodomain; WT, wild-type with endogenous aspartate-175.

**Figure 2:**
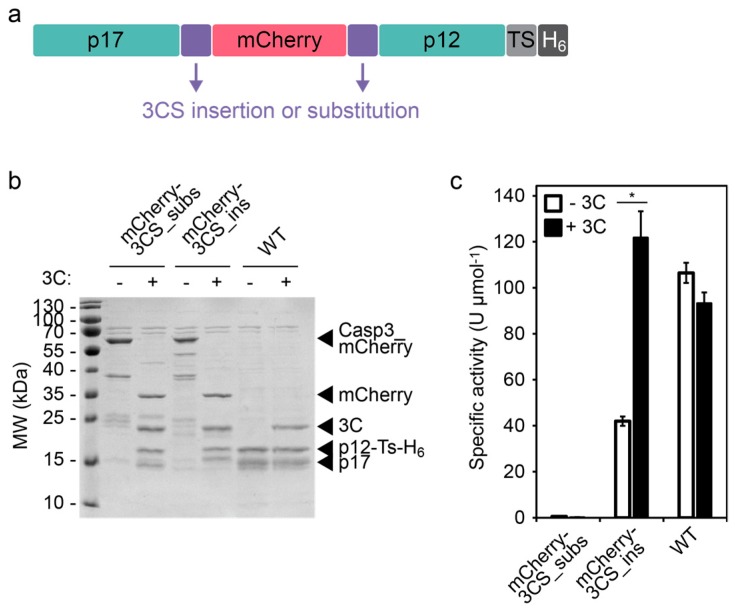
Incorporation of a bulky and 3C protease-removable protein domain into CASP3. (**a**) Schematic design of CASP3 with an incorporated fluorescent protein. The red fluorescent protein mCherry was positioned between the large (p17) and small (p12) subunits of CASP3. 3C protease cleavage sites (3CS) were incorporated either by the insertion or substitution strategy (see Figure 1). H_6_, hexa-histidine tag; TS, TEV protease cleavage site. (**b**) Cleavage of CASP3 constructs by 3C protease. The constructs mCherry-3CS_subs (3.9 µM), mCherry-3CS_ins (3.7 µM), and WT (6.4 µM) were incubated in the absence (−) or presence (+, 2.4 µM) of 3C protease (3C) for 4 h and analyzed using SDS-PAGE. (**c**) Activity of CASP3 constructs. The indicated constructs were incubated in the absence (−3C) or presence (+3C) of the 3C protease as described in (b). The specific activity of CASP3 in 1:100-diluted samples was determined using a synthetic peptide substrate. Mean values of three replicates +/− s.e.m. are shown. Statistics: unpaired t-test; * *p* < 0.05. Construct abbreviations: mCherry-3CS_subs, CASP3 with incorporated mCherry and substituted cleavage site; mCherry-3CS_ins, CASP3 with incorporated mCherry and inserted cleavage site; WT, wild-type CASP3.

To develop a CASP3 whose activity can be induced by the 3C protease but that is not capable of autoproteolytic activation, we applied two fundamental design strategies. The first strategy replaces the endogenous cleavage site between the large (p17) and small (p12) subunit with the 3C protease cleavage site (3CS, Figure 1), the second strategy separates the CASP3 subunits with a bulky protein domain that can be excised by the 3C protease (Figure 2).

### 2.1. Design and Testing of an Inducible CASP3 Via Endogenous Cleavage Site Engineering

To control the cleavage between p17 and p12 by the 3C protease, we replaced aspartate-175 (D175) at the P1 site of CASP3 by the 3CS (hereafter referred to as the “insertion” strategy, Figure 1a,b). Alternatively, we maintained the position of P1 and P1′ by substituting amino acids 170–177 with the 3CS (in the following referred to as “substitution” strategy, Figure 1a,b). The role of the prodomain of CASP3 is not yet fully understood. In the cellular context, it has been suggested that the prodomain is involved in the regulation of CASP3 activity and CASP3 mutants lacking the prodomain exhibit a lower activation threshold [21]. Therefore, we included a construct without the N-terminal prodomain in our study.

We produced the CASP3 constructs in *Escherichia coli* (*E. coli*) and purified them via a C-terminal hexa-histidine tag (His-tag) using affinity chromatography. We incubated the constructs in the presence or absence of the 3C protease and analyzed the cleavage products using sodium dodecyl sulfate polyacrylamide gel electrophoresis (SDS-PAGE, Figure 1c). In the absence of 3C protease, all CASP3 constructs remained in their full-length form, confirming their inability to autoprocess. By contrast, addition of 3C protease completely cleaved the constructs into the p17 and p12 fragments (Figure 1c). It should be noted that the p12 fragments run at a higher molecular weight compared to p17 because of a C-terminal extension (6 kDa) comprising a TEV-removable His-tag. Moreover, the insertion of the eight-amino acid 3CS results in a p17 fragments with higher molecular weight compared to the construct with substituted cleavage site (Figure 1c). 

Next, we evaluated the activity of the constructs using a commercially available synthetic substrate (DEVD-*p*NA, Figure 1d). In the absence of 3C protease, the engineered caspases did not show any measurable activity under the assay conditions. In the presence of 3C protease, however, both constructs with inserted 3CS (3CS_ins) exhibited proteolytic activities in the range of the wild-type CASP3 (WT). By contrast, the construct with the substituted cleavage site (3CS_subs) could not be activated by the 3C protease despite effective proteolytic processing (Figure 1d). These results indicate the importance of the endogenous residues between amino acids 170 and 177 that have been deleted for the cleavage site substitution.

### 2.2. Design and Testing of an Inducible Caspase-3 by Inserting a Bulky Protein Domain

The second design strategy separates p17 and p12 by a bulky protein domain (here: the red fluorescent protein mCherry), which prevents CASP3 from adopting its active conformation (Figure 2a). We inserted a 3CS at the C- and N-terminus of p17 and p12, respectively, to be able to excise mCherry and control the formation of active heterotetramers. Also, with these constructs, we compared the 3CS insertion with the substitution design. SDS-PAGE analysis showed that only in the presence of 3C protease the engineered CASP3 proteins were cleaved into the p17, p12, and mCherry fragments (Figure 2b). The wild-type CASP3 was autoproteolytically cleaved irrespective of the presence or absence of the 3C protease. 

In agreement with the above described results (Section 2.1), evaluation of the proteolytic activities revealed that the activity of CASP3 could only be induced by an inserted 3CS but not with a substituted cleavage site. The 3C protease-activated CASP3 (mCherry-3CS_ins) cleaved the synthetic peptide with a similar activity as the wild-type CASP3. However, the construct showed a comparably high basal activity (approximately 40% of the wild-type construct) in the absence of 3C protease, indicating structural flexibility and the ability of non-cleaved constructs to partially adopt an active conformation.

For further experiments, we used the inducible CASP3 with inserted 3CS (3CS_ins, Figure 1) because it showed the highest dynamic range with no detectable basal activity.

### 2.3. Inducible CASP3 as a Research Tool

The application potential of proteases is broad and ranges from addressing biotechnological and synthetic biological challenges to the tackling of basic research questions. We used our inducible CASP3 (3CS_ins) to validate this dual benefit in applied and fundamental research. For this, we (i) designed a three-tiered protease cascade and (ii) characterized effects of CASP3 mutations.

#### 2.3.1. Control of Proteolytic Cascades

Proteases represent popular enzymes for the engineering of artificial signal transduction systems [5,7,24,25,26]. For this, the spatial and temporal control of protease activity is critical. This can be achieved by, for example, controlling the temporal availability of inducers and immobilizing the proteins on solid supports.

**Figure 3:**
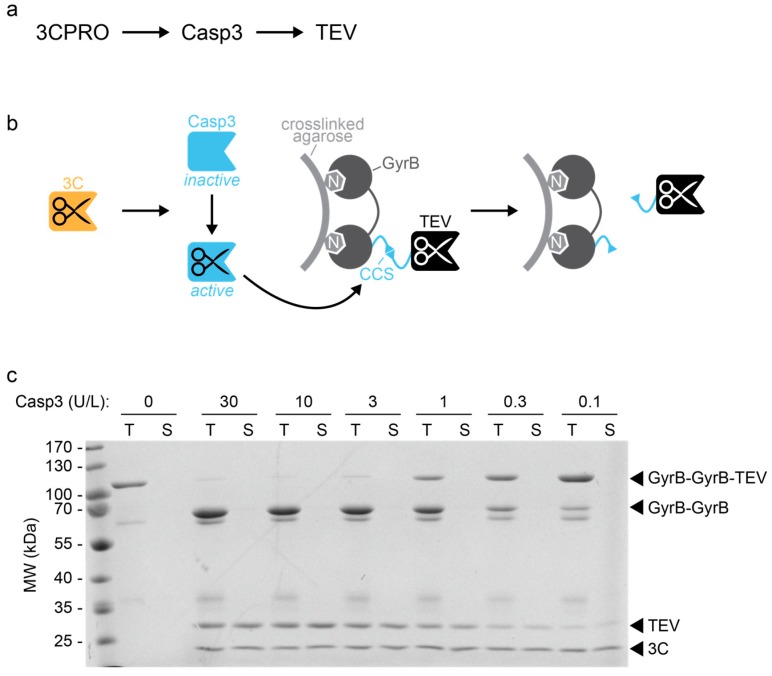
Engineering of a three-tiered protease cascade. (**a**) Sequence of the protease cascade comprised of the 3C protease (3CPRO), CASP3, and TEV protease. (**b**) Schematic representation of the design. The TEV protease is fused via a CASP3-cleavable linker (CASP3 cleavage site, CCS) to a bacterial gyrase subunit B (GyrB) tandem for its immobilization to novobiocin (N)-functionalized agarose. In the presence of 3C protease (3C), CASP3 is activated and triggers the release of the TEV protease from the solid support. (**c**) Testing of the designed protease cascade. An equivalent of 13 mg/mL TEV-agarose (corresponding to 2.2 µM TEV) was incubated in the absence of upstream proteases (lane 1 and 2) or in the presence of 3C protease (4.8 µM) and the indicated concentrations of inducible CASP3 (3CS_ins; lane 3–14; 1 U/L corresponded to 0.017 µM CASP3) overnight at RT. The cleavage and release of the TEV construct was evaluated using SDS-PAGE analysis of the supernatant (S) or the total protein (T, protein in the supernatant and on the material).

Here, we apply these principles for the design of a three-tiered protease cascade (Figure 3a). The cascade is initiated by the 3C protease, which cleaves and activates an inducible CASP3. Active CASP3 then triggers the release of the TEV protease from a solid support (Figure 3b).

To immobilize the TEV protease on a solid support, we utilized the interaction between the aminocoumarin antibiotic novobiocin and the bacterial gyrase subunit B (GyrB). We covalently coupled novobiocin to crosslinked agarose and fused the TEV protease via a CASP3-cleavable linker to a GyrB tandem (Figure 3b). Addition of the 3C protease induces the activation of CASP3 and thus the cleavage of the TEV linker. As a result, the TEV protease is released from the solid support.

We tested the functionality of the cascade by analyzing the release of TEV in response to different CASP3 (3CS_ins) concentrations. For this, we evaluated the proteins in the supernatant as well as the total protein in the system using SDS-PAGE (Figure 3c). In the absence of upstream proteases, the TEV construct was not cleaved, remained immobilized on the solid support, and thus was not detectable in the supernatant. Addition of the upstream proteases led to a CASP3 concentration-dependent cleavage and release of the TEV protease. By contrast, the GyrB domains remained coupled to the support. These results indicate the functionality of the protease cascade and illustrate an example of a pathway motif that could be incorporated into artificial signaling circuits [5,7,24,25].

#### 2.3.2. Characterizing the Effect of CASP3 Mutations

The control of enzymatic activity offers possibilities to investigate the impact of mutations on enzyme function. We focused our analysis on aspartate mutations in the large and small subunit of CASP3. Aspartate-169 (D169) lies close to the catalytic cysteine (C163) and its mutation to alanine (D169A) results in lack of automaturation [27]. Likewise, D192 is required for the catalytic activity of CASP3 and mutation of this position to alanine (D192A) completely abolishes the capability to remove the prodomain and thus the generation of the p17 large subunit, even upon processing by granzyme B (GrB) [28]. In contrast, mutation of the safety catch residues D179-181 to alanine or glutamate results in accelerated automaturation or a wild-type phenotype, respectively [19].

**Figure 4:**
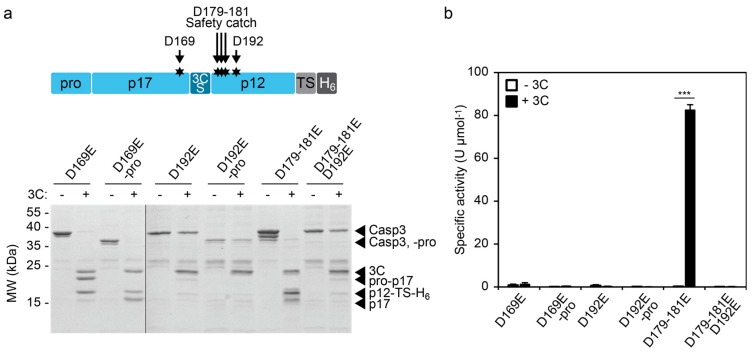
Effects of aspartate mutations on cleavage and activity of the inducible CASP3 (3CS_ins). (**a**) 3C protease-mediated cleavage of aspartate-to-glutamate CASP3 (3CS_ins) mutants. The indicated CASP3 constructs (5.2 µM or 5.7 µM of the constructs with or without prodomain, respectively) were incubated in the absence (−) and presence (+, 2.4 µM) of 3C protease (3C) for 4 h at RT and evaluated using SDS-PAGE. (**b**) Activity of mutant CASP3 (3CS_ins) constructs. The indicated CASP3 mutants were incubated with (+3C) or without (−3C) 3C protease as described in (a). Specific activities were assessed using a colorimetric assay using 1:100-diluted samples and a synthetic peptide substrate. Mean values of three replicates +/− s.e.m. are shown. Statistics: unpaired t-test; *** *p* < 0.005.

We mutated these aspartate positions in our inducible CASP3 (3CS_ins) to glutamate residues and investigated the impact on 3C protease-mediated cleavage and activation (Figure 4).

CASP3 (3CS_ins)-D169E was efficiently cleaved by 3C protease (Figure 4a). However, the construct showed, irrespective of the presence of the 3C protease, no detectable CASP3 activity as evidenced by its inability to remove the prodomain in *cis* (Figure 4a) or to cleave the DEVD-*p*NA substrate in *trans* (Figure 4b). In agreement with the previously described effects of CASP3-D169A [27], these results indicate the importance of D169 for proper catalytic function of the enzyme. Similarly, the mutant CASP3 (3CS_ins)-D192E showed no enzyme activity after cleavage by the 3C protease (Figure 4b). Interestingly, SDS-PAGE analysis revealed a low cleavage efficiency from the 3C protease indicating structural hindrance toward reaching the cleavage site within CASP3 (3CS_ins)-D192E (Figure 4a). Crystal structure analyses of cleaved CASP3 have suggested the formation of loop bundle interactions between amino acids 176–192 and loops of the second heterodimer comprising the active site as well as the catalytic cysteine [27]. These interactions stabilize the active conformation of cleaved CASP3. The structural importance of residues involved in the loop bundle interactions is in agreement with our results, which indicate structural changes of CASP3 (3CS_ins)-D192E that render the 3CS inaccessible. On the other hand, mutation of the safety catch residues D179-181 to glutamate did not influence enzymatic function. The construct was efficiently cleaved by 3C protease, which correlated with a specific activity in the range of the wild-type construct (see Figure 1d and Figure 4b). Also, in this D179–181E background, mutation of D192 to glutamate blocked the accessibility of the activating cleavage site and completely abolished enzyme function (Figure 4).

In summary, these results suggest the usability of our inducible CASP3 to evaluate structural and functional effects caused by aspartate mutations. In a broader perspective, inducible proteases or enzymes may offer high potential for insights into their molecular function.

## 3. Discussion

Over the last decade, proteases have become pivotal players in various fields of research. In this study, we designed 3C protease-inducible caspases as a flexible tool in, for example, synthetic biology, biotechnology, and fundamental research. We tested different design concepts and compared the replacement of the endogenous aspartate-175 with the 3CS (termed “insertion”) with the substitution of residues 170–177 with the 3CS (termed “substitution”). Whereas the insertion strategy resulted in 3C protease-inducible CASP3 constructs, the substitution rendered CASP3 inactive. Kang et al. previously described the development of an activatable CASP3 by substituting residues 172–177 with a thrombin cleavage site [20]. Thus, it can be assumed that the cysteine and/or glycine at position 170 and 171, respectively, play a crucial role for CASP3 activity. These amino acids lie in the active site loop L2 comprising residues 163–175 and thus near the catalytic cysteine (C163, Figure 1b). This suggests that amino acids close to C163 may be more critical for the correct positioning of the catalytic triad than the C-terminal residues of loop L2.

As a complementary approach, we aimed to sterically block the formation of active heterotetramers by inserting a bulky protein domain between p17 and p12. Indeed, inserting the fluorescent protein mCherry decreased the basal activity of CASP3. However, the construct retained considerable residual activity (approximately 40% of the wild-type construct). Although the mCherry domain negatively influenced the formation of the fully active conformation, it likely decreased the structural constraints of loops L2 and L2′ too (residues 176′–192′ from the second heterodimer [27]). As a consequence, the increased flexibility seemed to facilitate the formation of the active loop bundle. These results are in agreement with a previous report showing that the insertion of long and flexible linkers increased the activity of CASP3 as a function of the linker length [9]. The general design rules that can be deduced from these data include the consideration of the site of insertion (replacement of only D175 versus substitution of additional amino acids that might affect CASP3 activity) as well as the size, structure, and flexibility of inserts.

We validated the application potential of our 3C protease-inducible CASP3 (3CS_ins) by designing a three-tiered protease cascade. The protease cascade was initiated by the 3C protease and led to the CASP3-mediated release of TEV protease from a solid support. The amount of TEV protease that was cleaved off and released into the supernatant was dependent on the concentration of CASP3 (3CS_ins). This proof-of-concept release system represents a basic design motif that can be incorporated into more complex artificial signal sensing and transducing systems [4,5,24,25,26]. Complementary and orthogonal proteases that can be induced by other proteases [4,5], peptides [5], small molecules [8], or light [9,10] could be used to extend such systems to higher-order networks. Moreover, affinity and scaffolding functionalities can be fused to engineered proteases to generate multi-enzyme complexes. Recently, Stein et al. applied this principle for the design of protease cascade-based ultrasensitive sensors [29]. The engineering of protease-based circuits has paved the way for synthetic biology-inspired systems capable of autonomously sensing and processing distinct input stimuli both in cells and in vitro. This includes, for example, the engineering of binary logic gates [4,7], bandpass filters [7], and signal-amplifying [5,24,25,29], as well as light-pulse-counting circuits [26]. Such information-processing systems hold promise for analytical, therapeutic, and biotechnological applications.

In addition to the bottom-up design of synthetic enzyme circuits, we tested the impact of aspartate mutations on catalytic activity. Whereas the CASP3 (3CS_ins)-D169E mutant was efficiently cleaved by the 3C protease, the CASP3 (3CS_ins)-D192E mutant showed limited accessibility for cleavage. D169 lies close to the catalytic cysteine within the L2 loop and might influence the alignment of the catalytic triad, similar to residues 171 and 172. In contrast, D192 is located in another loop (L2′), which does not interact with the active site loops in *cis*, but is involved in interactions within the loop bundle of the second heterodimer. Our results indicate that the D192E mutation might not only impair the formation of the active loop bundle but also the overall structure of CASP3. This observation hints at a crucial structural role of D192 and mutation of this residue to glutamate is sufficient to mask the interdomain linker of CASP3. Consequently, the mutant was neither able to remove its prodomain nor to cleave a synthetic peptide in *trans*. A previous study on CASP3-D192A has shown that also the aspartate-to-alanine mutation completely abolishes catalytic activity [28]. Neither human GrB nor caspase-8 were able to activate CASP3-D192A, as evidenced by the failure of autocatalytically generating the p17 fragment. Consistent with our results, the cleavage efficiency of CASP3-D192A by caspase-8 was impaired. Nevertheless, human GrB was still able to cleave CASP3. This suggests that, despite the structural changes caused by mutated D192, the cleavage site might not be generally blocked for all proteases. However, further studies are required to assess whether the degree of sterical hindrance of D192E and D192A mutants is similar. Regardless of the cleavage efficiency, the aspartate at position 192 seems to be essential for the catalytic activity of CASP3. Mutation of this residue to asparagine has been found in the primary tumor tissue of a patient with endometrioid carcinoma [30] indicating the medical significance of this amino acid position.

Taken together, this work illustrates design strategies of 3C protease-inducible CASP3 constructs and highlights the applicability of engineered proteases for synthetic biological release systems, as well as fundamental research questions. In a broader perspective, the herein applied concept could also be applied to engineer and study other initiator and effector caspases, or zymogens in general.

## 4. Materials and Methods

### 4.1. Plasmids

All plasmids were constructed using Gibson assembly [31], except those coding for the single point mutation constructs (D169E and D192E), which were generated using site-directed mutagenesis of the 3CS_ins construct. Details about the cloning of the constructs are given in Table S1 and Table S2 of the Supplementary Material.

### 4.2. Protein Production and Purification

All proteins were produced in BL21(DE3)pLysS *E. coli* (Thermo Fisher Scientific, Waltham, MA, USA, # C602003). Bacteria were cultivated in shake flasks containing LB medium supplemented with 100 µg mL^−1^ ampicillin and 36 µg mL^−1^ chloramphenicol. At an OD_600_ of 0.6, gene expression was induced by 1 mM isopropyl β-d-1-thiogalactopyranoside (IPTG) at 37 °C for 4 h. Bacterial cells were harvested at 6000× *g*, resuspended in Ni lysis buffer (50 mM NaH_2_PO_4_, 300 mM NaCl, 10 mM imidazole, pH 8.0), and lyzed via sonication (Bandelin Sonopuls HD 3100 homogenizer, BANDELIN, Berlin, Germany). Cellular debris was removed using centrifugation at 30,000× *g* for 30 min. Supernatants were subjected to immobilized metal affinity chromatography (IMAC) using Ni^2+^-nitrilotriacetic acid (Ni-NTA)-agarose (QIAGEN, Hilden, Germany, #30230) as described previously [24]. Proteins were eluted in Ni elution buffer (50 mM NaH_2_PO_4_, 300 mM NaCl, 250 mM imidazole, pH 8.0) and supplemented with 10 mM 2-mercaptoethanol (2-ME). Protein concentrations were determined using a Bradford assay (protein assay dye reagent, Bio-Rad, Hercules, CA, USA, #5000006) using bovine serum albumin (BSA, Sigma Aldrich, St. Louis, MO, USA, #05479) as protein standard. 3C protease was supplemented with 10% (*v*/*v*) glycerol and stored at −80 °C until use.

### 4.3. Cleavage and Analysis of CASP3 Constructs

A total of 10–12 µg caspase was incubated with 2.5 µg 3C protease in 50 µL Ni elution buffer supplemented with 10 mM 2-ME for 4 h at RT. The cleavage reactions were evaluated using SDS-PAGE (15% (*w*/*v*) gels) and Coomassie brilliant blue staining (Carl Roth, Karlsruhe, Germany, #3862). CASP3 activity was analyzed using a colorimetric CASP3 assay using the synthetic peptide DEVD-*p*NA as a substrate (BioVision, Milpitas, CA, USA, #1008). For this, the samples were diluted 1:100 in a 50 µL Ni elution buffer supplemented with 10 mM 2-ME. The CASP3 assay was conducted in transparent, flat-bottom 96-well plates (Carl Roth, Karlsruhe, Germany, #9293.1) by mixing 30 µL CASP3 dilutions with 2 × 30 µL reaction buffer, supplemented with 10 mM dithiothreitol (DTT) and 0.3 mM DEVD-*p*NA, and monitoring the absorbance at 405 nm every minute for at least 30 min. A dilution series of *p*-nitroaniline (0–500 mM) was used as the calibration standard.

### 4.4. Protease Cascade

Novobiocin-coupled crosslinked agarose was synthesized as previously described [24]. The binding capacity of the material (0.17 nmol functional groups per gram agarose) was determined elsewhere [32]. To immobilize TEV on the material, 1 nmol TEV per 1 mg novobiocin-functionalized agarose was incubated in protease buffer (50 mM NaH_2_PO_4_, 300 mM NaCl, 10% (*v*/*v*) glycerol, 5 mM 2-ME, pH 8.0) at 4 °C overnight with agitation. The TEV-coupled material was washed with protease buffer to remove unbound protein and suspended in protease buffer. An equivalent of 13 mg/mL TEV-agarose (corresponding to 1 µM TEV) was supplemented with 3C protease (0–5 µg) and the indicated CASP3 (3CS_ins) concentrations and incubated at room temperature overnight. The cleavage and release of the TEV construct was evaluated using SDS-PAGE (12% (*w*/*v*) gel).

### 4.5. Statistics

Unpaired t-tests assuming unequal variances were conducted using the data analysis tool of Microsoft Excel 2013 (version no. 15.0.5119.1000); *** *p* < 0.005, * *p* < 0.05.

## Supplementary Materials

The following are available online at https://www.mdpi.com/1420-3049/24/10/1945/s1, Table S1: Plasmids used in this study, Table S2: Oligonucleotides used in this study.

## Abbreviations

3CS_ins	CASP3 with inserted 3C protease cleavage site
3CS_ins -pro	3CS_ins with deleted prodomain
3CS_subs	CASP3 with substituted cleavage site
3CS_subs -pro	3CS_subs with deleted prodomain
3CS	HRV14 3C protease cleavage site
BSA	Bovine serum albumin
CASP3	Human caspase-3
CCS	CASP3 cleavage site
DEVD-*p*NA	*p*-nitroaniline-labeled DEVD peptide for colorimetric CASP3 assay
DTT	Dithiothreitol
*E. coli*	*Escherichia coli*
GrB	Granzyme B
GyrB	Bacterial gyrase subunit B
His-tag	Hexa-histidine tag
HRV14	Human rhinovirus serotype 14
IMAC	Immobilized metal affinity chromatography
IPTG	Isopropyl β-d-1-thiogalactopyranoside
mCherry-3CS_ins	CASP3 with incorporated mCherry and inserted 3C protease cleavage site
mCherry-3CS_subs	CASP3 with incorporated mCherry and substituted cleavage site
2-ME	2-mercaptoethanol
Ni-NTA	Ni^2+^-nitrilotriacetic acid
p12	Small CASP3 subunit
p17	Large CASP3 subunit
SDS-PAGE	Sodium dodecyl sulfate polyacrylamide gel electrophoresis
TEV	Tobacco etch virus
TS	TEV protease cleavage site
WT	Wild-type
