# Supplementary material for: Design of a Human Rhinovirus-14 3C Protease-Inducible Caspase-3
Source: Molecules. 2019 May 21;24(10):1945. doi: 10.3390/molecules24101945 (PMC6571611; doi:10.3390/molecules24101945)
Supplement: Supplementary file 1 [file molecules-24-01945-s001.pdf]

molecules

Communication

Supplementary Information

Design of a Human Rhinovirus-14 3C Protease-Inducible Caspase-3

Hanna J. Wagner* and Wilfried Weber*

*Correspondence: hanna.wagner@biologie-uni-freiburg.de
wilfried.weber@biologie.uni-freiburg.de

Contents:

Table S1. Plasmids used in this study. 2

Table S2. Oligonucleotides used in this study. 5

References 6

molecules

MDPI

Table S1. Plasmids used in this study.

Plasmid	Description	Reference
pHJW1	P_{T7}-Casp3-TCS-His_6<br>Bacterial expression vector for the production of human Caspase-3 fused to a C-terminal linker region containing a TEV cleavage site (TCS) and a hexa histidine-tag (His_6).	[1,2]
pHJW4	P_{T7}-His_6-3CPRO<br>Bacterial expression vector for the production of His-tagged HRV14 3C protease.	[1,2]
pHJW14	P_{T7}-His_6-AviTag-GyrB-GyrB-CS-SNAP25(141-206)-TEV<br>Bacterial expression vector coding for the TEV protease fused to His- and Avi-tagged GyrB domains via a Casp3-cleavable linker and amino acids 141-206 of human SNAP25.	[3]
pHJW181	P_{T7}-Casp3(3CS_ins)-TCS-His_6<br>Bacterial expression vector encoding the Casp3 construct 3CS_ins (3C protease cleavage site inserted at position 175).<br>Amino acid sequence: prodomain, p17, 3CS, p12, TEV cleavable linker, His-tag<br>MMENTENSVDSKSIKNLEPKIIHGSESMDSGISLDNSYKMDYPEMGLCIIINNKNFHKSTGMTSRSGTDVDAAN<br>LRETFRNLKYEVRNKNDLTREEIVELMRDVSKEDHSKRSSFVCVLLSHGEEGIIFGTNGPVDLKKITNFFRGDRC<br>RSLTGKPKLFIIQACRGTELDCGIETLEVLFQGPSGVDDDMACHKIPVEADFLYAYSTAPGYYSWRNSKDGSWF<br>IQSLCAMLKQYADKLEFMHILTRVNRKVATEFESFSFDATFHAKKQIPCIVSMLTKELYFYSGGGSGGGGENLY<br>FQSGGGPAGEASSIPNREGKPIPNPLLGLGSTRTGEFHHHHHHH	[1,2]
pHJW187	P_{T7}- Δpro-Casp3(3CS_subs)-TCS-His_6<br>Bacterial expression vector encoding the Casp3 construct 3CS_subs -pro (residues 170 – 177 substituted by the 3C protease cleavage site).<br>The plasmid sequences were amplified from pHJW181 using oligonucleotides oHJW435 and oHJW436 (PCR 1), oHJW5 and oHJW437 (PCR 2), oHJW6 and oHJW205 (PCR 3). All three fragments were assembled by Gibson cloning [4].<br>Amino acid sequence: p17, 3CS, p12, TEV cleavable linker, His-tag<br>MSGISLDNSYKMDYPEMGLCIIINNKNFHKSTGMTSRSGTDVDAANLRETFRNLKYEVRNKNDLTREEIVELM<br>RDVSKEDHSKRSSFVCVLLSHGEEGIIFGTNGPVDLKKITNFFRGDRCRSLTGKPKLFIIQACRGTELDLEVLFQG<br>PVDDDMACHKIPVEADFLYAYSTAPGYYSWRNSKDGSWFIQSLCAMLKQYADKLEFMHILTRVNRKVATEFE<br>SFSFDATFHAKKQIPCIVSMLTKELYFYSGGGSGGGGENLYFQSGGGPAGEASSIPNREGKPIPNPLLGLGSTRT<br>GEFHHHHHHH	This work
pHJW188	P_{T7}-Δpro-Casp3(3CS_ins)-TCS-His_6<br>Bacterial expression vector for the production of the Casp3 construct 3CS_ins with deleted prodomain.<br>Plasmid fragments were amplified from pHJW181 using oligonucleotides oHJW5 and oHJW435 (PCR 1), and oHJW6 and oHJW205 (PCR 2), and assembled by Gibson cloning.<br>Amino acid sequence: p17, 3CS, p12, TEV cleavable linker, His-tag<br>MSGISLDNSYKMDYPEMGLCIIINNKNFHKSTGMTSRSGTDVDAANLRETFRNLKYEVRNKNDLTREEIVELM<br>RDVSKEDHSKRSSFVCVLLSHGEEGIIFGTNGPVDLKKITNFFRGDRCRSLTGKPKLFIIQACRGTELDCGIETLE<br>VLFQGPSGVDDDMACHKIPVEADFLYAYSTAPGYYSWRNSKDGSWFIQSLCAMLKQYADKLEFMHILTRVNR	This work

Plasmid	Description	Reference
	KVATEFESFSFDATFHAKKQIPCIVSMLTKELYFYSGGGSGGGGENLYFQSGGGPAGEASSIPNREGKPIPNPLL<br>GLGSTRTGEFHHHHHHH	
pHJW189	P_{T7}-Casp3(3CS_ins-D169E)-TCS-His_6<br>Bacterial expression vector encoding the Casp3 construct 3CS_ins with D169E mutation. The mutation D169E was inserted into 3CS_ins by site-directed mutagenesis of pHJW181 using oligonucleotides oHJW438 and oHJW439.	This work
pHJW190	P_{T7}-Casp3(3CS_ins-D192E)-TCS-His_6<br>Bacterial expression vector encoding the Casp3 construct 3CS_ins with D192E mutation. The mutation D192E was inserted into 3CS_ins by site-directed mutagenesis of pHJW181 using oligonucleotides oHJW440 and oHJW441.	This work
pHJW191	P_{T7}-Casp3(3CS_ins-D179-181E)-TCS-His_6<br>Bacterial expression vector coding for the Casp3 construct 3CS_ins with safety catch mutation (D179-181E).<br>The plasmid sequences were amplified from pHJW181 using oligonucleotides oHJW6 and oHJW442 (PCR 1), oHJW5 and oHJW443 (PCR 2). The resulting DNA fragments were assembled by Gibson cloning.	This work
pHJW192	P_{T7}-Casp3(3CS_ins-D179-181E, D192E)-TCS-His_6<br>Bacterial expression vector coding for the mutant Casp3 construct 3CS_ins with safety catch (D179-181E) and D192E mutation.<br>The plasmid sequences were amplified from pHJW181 using oligonucleotides oHJW6 and oHJW444 (PCR 1), oHJW5 and oHJW445 (PCR 2). The resulting DNA fragments were assembled by Gibson cloning.	This work
pHJW193	P_{T7}-Δpro-Casp3(mCherry-3CS_subs)-TCS-His_6<br>Bacterial expression vector for the production of Casp3 with deleted prodomain and incorporated mCherry; amino acids 170 – 177 of Casp3 were substituted by 3CS-mCherry-3CS.<br>The plasmid sequence was amplified from pHJW2 (Ref.: [1,2]) using oligonucleotides oHJW446 and oHJW447 (PCR 1), and from pHJW181 using oHJW435 and oHJW436 (PCR 2), oHJW5 and oHJW437 (PCR 3), and oHJW6 and oHJW205 (PCR 4). PCR fragments 1 and 2 were assembled by fusion PCR using oligonucleotides oHJW435 and oHJW447. The resulting fragment was assembled with PCR 3 and 4 by Gibson cloning.<br>Amino acid sequence: p17, 3CS, mCherry, p12, TEV cleavable linker, His-tag<br>MSGISLDNSYKMDYPEMGLCIIINNKNFHKSTGMTSRSGTDVDAANLRETFRNLKYEVRNKNDLTREEIVELM<br>RDVSKEDHSKRSSFVCVLLSHGEEGIIFGTNGPVDLKKITNFFRGDRCRSLTGKPKLFIIQACRGTELDLEVLFQG<br>PGGGSMAIIKEFMRFKVHMEGSVNGHEFEIEGEGEGRPYEGTQTAKLKVTKGGPLPFAWDILSPQFMYGSKAY<br>VKHPADIPDYLKLSFPEGFKWERVMNFEDGGVVTVTQDSSLQDGEFIYKVKLRGTNFPSDGPVMQKKTMGWE<br>ASSERMYPEDGALKGEIKQRLKLKDGGHYDAEVKTTYKAKKPVQLPGAYNVNIKLDITSHNEDYTIVEQYERA<br>EGRHSTGGMDELYKSGGGLEVLFQGPVDDDMACHKIPVEADFLYAYSTAPGYYSWRNSKDGSWFIQSLCAM<br>LKQYADKLEFMHILTRVNRKVATEFESFSFDATFHAKKQIPCIVSMLTKELYFYSGGGSGGGGENLYFQSGGGP<br>AGEASSIPNREGKPIPNPLLGLGSTRTGEFHHHHHHH	This work

Plasmid	Description	Reference
pHJW272	P_{T7}-Δpro-Casp3(3CS_ins-D192E)-TCS-His_6<br>Bacterial expression vector encoding the Casp3 construct 3CS_ins with D192E mutation and deleted prodomain.<br>The plasmid sequence was amplified from pHJW190 using oligonucleotides oHJW5 and oHJW435 (PCR 1), and oHJW6 and oHJW205 (PCR 2). The resulting DNA fragments were assembled by Gibson cloning.	This work
pHJW273	P_{T7}-Δpro-Casp3(3CS_ins-D169E)-TCS-His_6<br>Bacterial expression vector for the production of the Casp3 construct 3CS_ins with D169E mutation and deleted prodomain.<br>The plasmid sequence was amplified from pHJW189 using oligonucleotides oHJW5 and oHJW435 (PCR 1), and oHJW6 and oHJW205 (PCR 2). The resulting DNA fragments were assembled by Gibson cloning.	This work
pHJW274	P_{T7}-Casp3(mCherry-3CS_ins)-TCS-His_6<br>Bacterial expression vector for the production of Casp3 with deleted prodomain and incorporated mCherry; aspartate-175 was exchanged by 3CS-mCherry-3CS.<br>The plasmid sequence was amplified from pHJW2 (Ref.: [1,2]) using oligonucleotides oHJW447 and oHJW537 (PCR 1), and from pHJW1 using oHJW5 and oHJW538 (PCR 2), and oHJW6 and oHJW424 (PCR 3). The resulting fragments were assembled by Gibson cloning.<br>Amino acid sequence: p17, 3CS, mCherry, p12, TEV cleavable linker, His-tag<br>MMENTENSVDSKSIKNLEPKIIHGSESMDSGISLDNSYKMDYPEMGLCIIINNKNFHKSTGMTSRSGTDVDAAN<br>LRETFRNLKYEVRNKNDLTREEIVELMRDVSKEDHSKRSSFVCVLLSHGEEGIIFGTNGPVDLKKITNFFRGDRC<br>RSLTGKPKLFIIQACRGTELDCGIETLEVLFQGPGGGSMAIIKEFMRFKVHMEGSVNGHEFEIEGEGEGRPYEGT<br>QTAKLKVTKGGPLPFAWDILSPQFMYGSKAYVKHPADIPDYLKLSFPEGFKWERVMNFEDGGVVTVTQDSSL<br>QDGEFIYKVKLRGTNFPSDGPVMQKKTMGWEASSERMYPEDGALKGEIKQRLKLKDGGHYDAEVKTTYKAK<br>KPVQLPGAYNVNIKLDITSHNEDYTIVEQYERAEGRHSTGGMDELYKSGGGLEVLFQGPSGVDDDMACHKIP<br>VEADFLYAYSTAPGYYSWRNSKDGSWFIQSLCAMLKQYADKLEFMHILTRVNRKVATEFESFSFDATFHAKKQ<br>IPCIVSMLTKELYFYSGGGSGGGGENLYFQSGGGPAGEASSIPNREGKPIPNPLLGLGSTRTGEFHHHHHHH	This work

molecules

MDPI

Table S2. Oligonucleotides used in this study. Annealing sequences are underlined.

Oligo	Sequence (5′ → 3′)	Reference
oHJW1	CTTGATCCGGCTGCTAACAAAG	[1,2]
oHJW5	CTTTGATCTTTTCTACGGGGTCTG	[1,2]
oHJW6	GCGTCAGACCCCGTAGAAAAG	[1,2]
oHJW205	CTCCTTCTTAAAGTTAAACAAAATTATTTCTAGAGG	[1,2]
oHJW424	TGTCTCAATGCCACAGTCCAG	This work
oHJW435	GTTTAACTTTAAGAAGGAGATATACATATGTCTGGAATATCCCTGGACAACAG	This work
oHJW436	CCTGGAACAGAACTTCCAGGTCCAGTTCTGTACCACGGC	This work
oHJW437	GGAAGTTCTGTTCCAGGGGCCCGTTGATGATGACATGGCGTGTC	This work
oHJW438	GTGGTACAGAACTGGAGTGTGGCATTGAGACACTG	This work
oHJW439	CAGTGTCTCAATGCCACACTCCAGTTCTGTACCAC	This work
oHJW440	CATAAAATACCAGTGGAGGCCGAGTTCTTGTATGCATACTC	This work
oHJW441	GAGTATGCATACAAGAACTCGGCCTCCACTGGTATTTTATG	This work
oHJW442	CACGCCATCTCTTCTTCAACACCACTGGGCCC	This work
oHJW443	GAAGAAGAGATGGCGTGTCATAAAATACCAGTG	This work
oHJW444	CTCGGCCTCCACTGGTATTTTATGACACGCCATCTCTTCTTCAACACCACTGGGCCC	This work
oHJW445	CATAAAATACCAGTGGAGGCCGAGTTCTTGTATGCATACTCCACAGCAC	This work
oHJW446	CCTGGAAGTTCTGTTCCAGGGGCCCGGCGGCGGCAGCATGGCCATCATCAAGGAGTTC	This work
oHJW447	GGGCCCCTGGAACAGAACTTCCAGGCCGCCGCCAGACTTGTACAGCTCGTCCATGC	This work
oHJW537	GGACTGTGGCATTGAGACACTGGAAGTTCTGTTCCAGGGGCCCGGCGGCGGCAGCATGGCCATCATCAAGGAGTTC	This work
oHJW538	GTTCTGTTCCAGGGGCCCAGTGGTGTTGATGATGACATGG	This work

References

1. Wagner, H. J.; Engesser, R.; Ermes, K.; Geraths, C.; Timmer, J.; Weber, W. Synthetic biology-inspired design of signal-amplifying materials systems. *Mater. Today* **2019**, *22*, 25–34, doi:10.1016/j.mattod.2018.04.006.

2. Wagner, H. J.; Engesser, R.; Ermes, K.; Geraths, C.; Timmer, J.; Weber, W. Characterization of the synthetic biology-inspired implementation of a materials-based positive feedback loop. *Data in Brief* **2018**, *19*, 665–677, doi:10.1016/j.dib.2018.05.074.

3. Wagner, H. J.; Kemmer, S.; Engesser, R.; Timmer, J.; Weber, W. Biofunctionalized Materials Featuring Feedforward and Feedback Circuits Exemplified by the Detection of Botulinum Toxin A. *Adv. Sci.* **2019**, *6*, 1801320, doi:10.1002/advs.201801320.

4. Gibson, D. G.; Young, L.; Chuang, R.; Venter, J. C.; Hutchison, C. A.; Smith, H. O. Enzymatic assembly of DNA molecules up to several hundred kilobases. *Nat. Methods* **2009**, *6*, 343–5, doi:10.1038/nmeth.1318.
